# Oxidative stress-mediated epigenetic regulation by G-quadruplexes

**DOI:** 10.1093/narcan/zcab038

**Published:** 2021-09-16

**Authors:** Aaron M Fleming, Cynthia J Burrows

**Affiliations:** Department of Chemistry, University of Utah, 315 S. 1400 East, Salt Lake City, UT 84112-0850, USA; Department of Chemistry, University of Utah, 315 S. 1400 East, Salt Lake City, UT 84112-0850, USA

## Abstract

Many cancer-associated genes are regulated by guanine (G)-rich sequences that are capable of refolding from the canonical duplex structure to an intrastrand G-quadruplex. These same sequences are sensitive to oxidative damage that is repaired by the base excision repair glycosylases OGG1 and NEIL1–3. We describe studies indicating that oxidation of a guanosine base in a gene promoter G-quadruplex can lead to up- and downregulation of gene expression that is location dependent and involves the base excision repair pathway in which the first intermediate, an apurinic (AP) site, plays a key role mediated by AP endonuclease 1 (APE1/REF1). The nuclease activity of APE1 is paused at a G-quadruplex, while the REF1 capacity of this protein engages activating transcription factors such as HIF-1α, AP-1 and p53. The mechanism has been probed by *in vitro* biophysical studies, whole-genome approaches and reporter plasmids *in cellulo*. Replacement of promoter elements by a G-quadruplex sequence usually led to upregulation, but depending on the strand and precise location, examples of downregulation were also found. The impact of oxidative stress-mediated lesions in the G-rich sequence enhanced the effect, whether it was positive or negative.

## Graphical Abstract

**Graphical Abstract:**
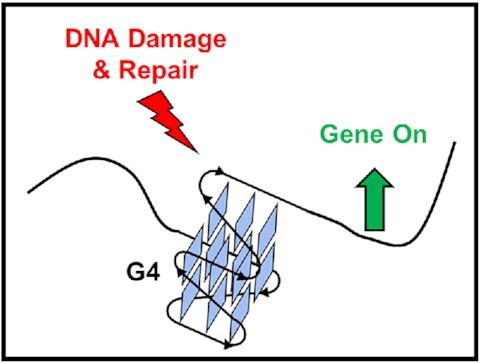
Guanine-rich sequences in promoter regions of genes are sensitive to oxidative stress and may refold to G-quadruplexes. The DNA repair process impacts gene expression through the ability of AP-endonuclease to bind and recruit activating transcription factors.

## INTRODUCTION

Many cancer-associated genes are regulated by guanine (G)-rich sequences in their gene promoters through the formation of G-quadruplex (G4) secondary structures (1). These sites also represent hotspots for oxidative damage (2), and oxidative stress is a feature common to many cancer types (3). Thus, the intersection of G oxidation and G4 folding in promoter regions can have a major impact on gene expression and consequently on the progression of cancer (4). Here, we summarize the major pathways of G oxidation, its impact on G4 folding and what we know so far about the role of DNA repair in modulating gene expression when DNA damage is present in a potential G4 sequence (PQS).

**Figure 1.**
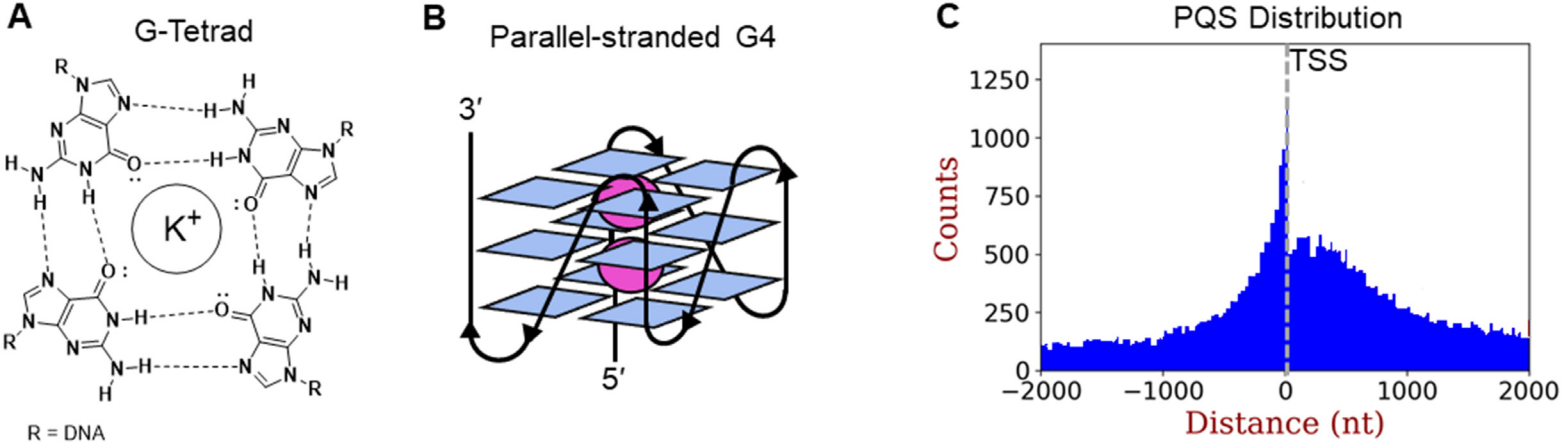
(**A**) The G-tetrad forms one layer of a folded G4. (**B**) A parallel G4. (**C**) Distribution of PQSs with respect to the TSS (11).

G4s can form from DNA or RNA sequences containing four or more closely spaced G tracks comprised of G_*n*≥3_ for DNA or G_*n*≥2_ for RNA in which one G from each of four tracks is hydrogen bonded in Hoogsteen fashion to create a flat G tetrad; these then assemble via π stacking with K^+^ ions bound in central positions in between layers (Figure 1A and B) (5). Although the human telomere sequence (hTelo) exhibits a more complex fold, adopting parallel, antiparallel and hybrid folds with cation dependence, most G4 structures in DNA gene promoters and in RNA are parallel stranded with all of the G tracks oriented in the same direction yielding a compact structure with extended flat surfaces above and below the stacked quartets (6). The core is held together by loops, comprised of typically one to three nucleotides, that bridge from the top of the stack to the bottom. Interestingly, this is most commonly achieved with only one nucleotide in the loop. Longer loops can favor antiparallel arrangements of the G tracks and may also form hairpins or more complex structures (7,8). Myriad examples have been analyzed by NMR spectroscopy that include bulged nucleotides or other unusual secondary structures (7,8). Generally speaking, cationic small molecules with extended aromatic rings are likely to be candidates for G4 binding, and the diversity of 3D structures provided by the loops and flanking sequences lends hope to the idea of G4-specific targeting for therapeutic applications (9,10).

PQSs exist in hundreds of thousands of sites in the human genome, but their distribution is non-random (12). Apart from telomeres at the ends of chromosomes, PQSs are common in regulatory regions in mammalian and plant genomes but less common in bacteria and yeast (13,14). In mammals, the intron-1/exon-1 boundary has an unusually high frequency of PQSs, where the sequence may serve as a site for *N*^6^-methylation of adenosine in pre-mRNA and regulation of mRNA splicing (15,16). Of interest to our laboratory is the high frequency of PQSs in gene promoters, just in advance of the transcription start site (TSS) (17,18). Pertinent to this discussion, we found that many of the 191 genes involved in mammalian DNA repair have PQSs in their promoters, and that their distribution was similar to the overall set of ∼10 000 genes that harbor G4s (Figure 1C) (17,19). Notably, many oncogenes and other cancer-associated genes (e.g. *c-MYC*, *VEGF*; see Table 1) have G4 sequences that appear to regulate their expression making these sites targets for pharmaceutical intervention (1,20). For DNA repair genes, the prevalence of G4s makes sense if the G4 structure is sensitive to the presence of chemical modifications from oxidation, alkylation, deamination or strand breaks. For oxidation, G4 folding during endogenous oxidative stress can upregulate the repair genes needed for correcting lesions in the genome. Additionally, 5′-untranslated regions (5′-UTRs) just downstream of the TSS often contain PQSs, and if present in the non-template strand of DNA, they would be transcribed to form PQSs in the 5′-UTR of mRNA, presenting two opportunities to regulate gene expression either in DNA or in RNA (17,18). Furthermore, mounting evidence suggests that G4s near the TSS, which can be a difficult site to pinpoint, can participate in R-loop formation wherein the DNA–RNA hybrid plays a role in gene initiation (21,22). In this situation, the presence of folded G4s in the non-template strand helps keep the two strands separated and may assist in the recruitment of factors needed for RNA synthesis and elongation (23).

**Table 1.** Examples of G4-forming sequences in the human genome and their locations

Gene/name	Strand	Location w.r.t. TSS	Sequence (5′ to 3′)	Reference
hTelo	n.a.	n.a.	TA**GGG**TTA**GGG**TTA**GGG**TTA**GGG**TTA**GGG**TT…	(24)
*VEGF*	Non-template	−50	CC**GGGG**C**GGG**CC**GGGGG**C**GGGG**TCCCGGC**GGGG**TC	(25)
*c-MYC*	Template	−262	CC**GGG**A**GGGG**CGCTTAT**GGGG**A**GGG**T**GGGG**A**GGG**T**GGGG**AAGGT**GGGG**AG	(26)
*KRAS*	Template	−104	GA**GGG**AGCGGCTGA**GGG**CGGTGT**GGG**AAGA**GGG**AAGA**GGGGG**AG	(27)
*HIF-1α*	Template	−48	TC**GGG**CGCGC**GGGG**A**GGGG**AGA**GGGGG**C**GGG**AGC	
*HSP90*	Non-template	−77	GA**GGG**C**GGG**CCAAA**GGG**AA**GGGG**T**GGG**CT	(28)
*TERT*	Template	−18	AA**GGGG**A**GGGG**CT**GGG**A**GGG**CCCGGA**GGGGG**CT**GGG**CC**GGGG**ACCC**GGG**A**GGGG**TC**GGG**AC**GGGG**C**GGGG**TC	(29)
*BCL2*	Template	−1386	GA**GGGG**C**GGG**CGC**GGG**AGGAA**GGGGG**C**GGG**AGC**GGGG**CT	(7)
*NEIL3*	Non-template	−3	TA**GGG**TGCTGTTT**GGG**C**GGGG**CCT**GGG**C**GGGG**CC	(30)
*NTHL1*	Non-template	−279	TC**GGG**TTGCAGT**GGG**CGC**GGG**TGA**GGG**CCC**GGG**AC	(31)
*PCNA*	Non-template	−126	CA**GGG**AGGCA**GGG**CGAC**GGGGG**C**GGGG**C**GGGG**CG	(32)
*RAD17*	Template	−18	CC**GGG**A**GGG**ACT**GGG**CT**GGGG**CAGGCT**GGGG**CG	(33)

### DNA damage and repair as a trigger for G4 folding

Hypoxia, metabolic levels and inflammation intertwine in cells to generate oxidative stress—an imbalance of oxidants (O_2_) and reductants [thiols and NAD(P)H] (34). Reactive oxygen species (ROS) are generated from both very low [O_2_] and high [O_2_] conditions and can take many forms. Although the Fenton reaction is invoked as a cellular source of hydroxyl radical (HO^•^), this reaction is intercepted by even low concentrations of bicarbonate, a major buffering component of mammalian cells, so that Fe(II) complexes reacting with H_2_O_2_ produce CO_3_^•−^ as a major ROS species instead of HO^•^ (35,36). Carbonate radical anion is also formed during inflammation in which peroxynitrite (from upregulated iNOS—inducible nitric oxide synthase) reacts with dissolved CO_2_ to yield ultimately CO_3_^•−^ again as the principal ROS (37). The distinction between HO^•^ and CO_3_^•−^ is important because these two radicals react very differently with DNA. Hydroxyl radical is highly reactive and abstracts H^•^ indiscriminately from all sites in 2-deoxyribose as well as forming adducts with all DNA and RNA bases (38). Ionizing radiation can also directly form base radicals by electron ejection as well as forming HO^•^ (39); however, we have proposed that *endogenous* pathways, via peroxynitrite- or Fe(II)-mediated oxidation of HCO_3_^−^/CO_2_, tend to favor the formation of CO_3_^•−^ as the major ROS attacking DNA in aerobic organisms (35,36).

**Figure 2.**
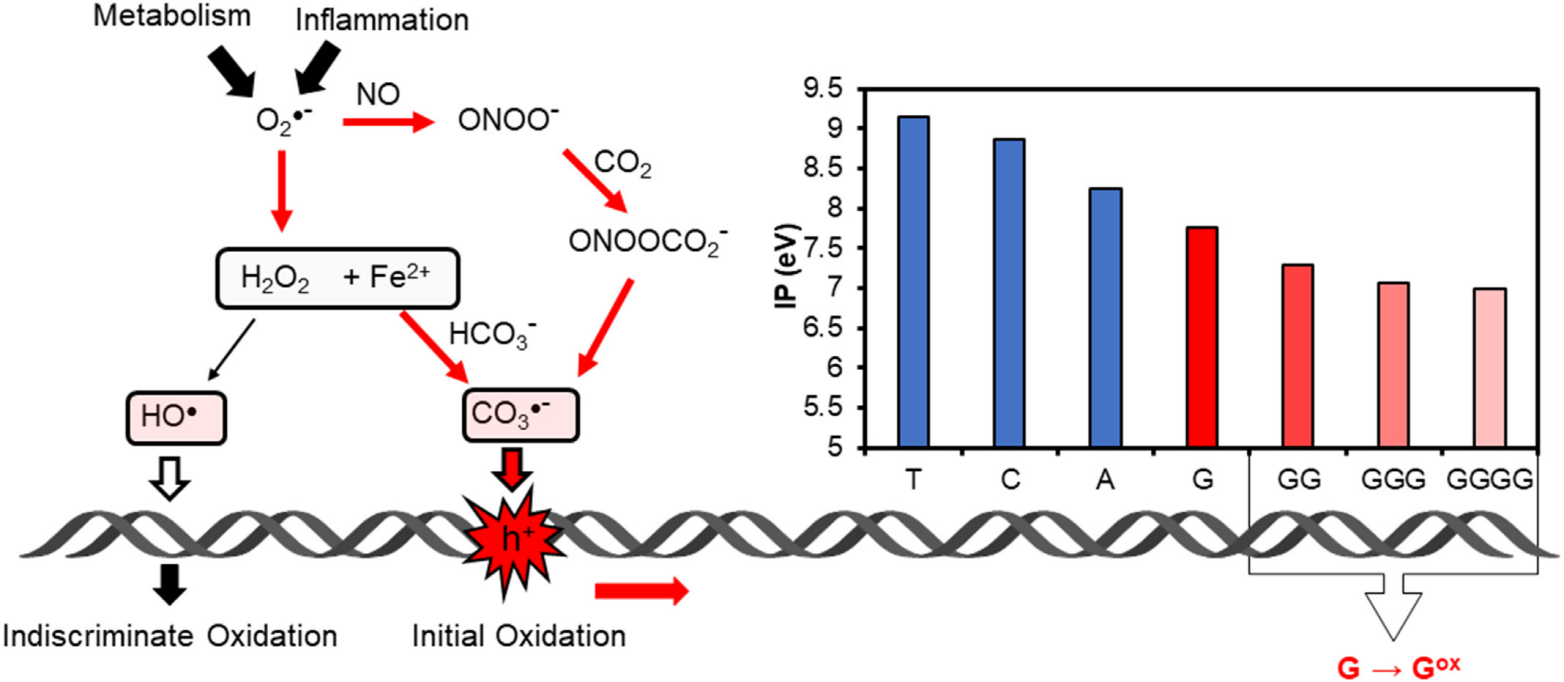
ROS, principally CO_3_^•−^ from endogenous oxidative stress, generates electron holes in DNA that travel the duplex looking for a low ionization potential site such as a G track of a PQS (4).

**Figure 3.**
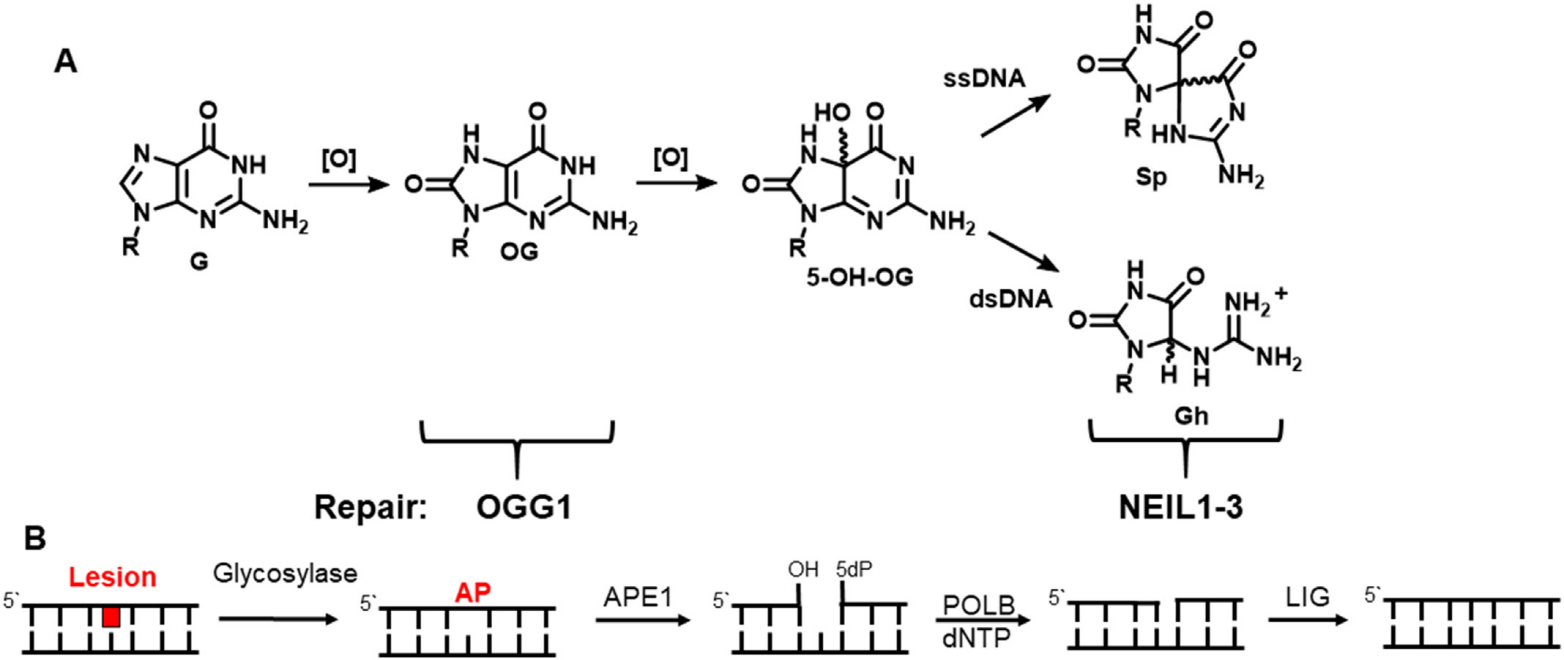
(**A**) Oxidation products of G include OG, the most common, and its overoxidation products Sp and Gh. (**B**) These lesions are repaired by the BER pathway using OGG1 for OG and NEIL glycosylases for Sp and Gh.

In contrast to HO^•^, CO_3_^•−^ acts as a one-electron oxidant and is more selective in its reactions (40). Because G is the most electron-rich of the nucleobases, and correspondingly has the lowest reduction potential (Figure 2), it is the most common nucleotide to be modified during oxidative stress (40). The immediate product of electron loss is a guanosine radical cation, G^•+^, which represents an electron hole. In DNA that is perfectly base paired, the double helix can easily transport electron holes along the π-stacked bases until the most stable site is found (41,42). Following this rapid charge transport, which has been demonstrated to occur over hundreds or even thousands of base pairs (43), a slower chemical step produces the oxidized base, typically 8-oxo-7,8-dihydro-2′-deoxyguanosine (OG, Figure 3) (38). The most stable site for the G^•+^ is one in which one or more G bases are stacked on its 3′ side. Thus, the G tracks of a PQS, i.e. 5′-GGG-3′, in its duplex state, are the best sites for one-electron oxidation by CO_3_^•−^ directly, or indirectly via charge transport (41,42). In such a sequence, any G followed by a 3′ G will be a good site for oxidation; thus, underlined Gs in the sequence above would be most susceptible to form OG.

Folded G4s are also targets for oxidation, but the pattern of reactivity is somewhat different. Among the π-stacked Gs of a 5′-GGG-3′ track, there is a distinct preference for oxidation of the 5′-most G (44). We speculate that the central G, which would normally follow the rule of being highly reactive in a duplex because of the presence of the 3′ G, is less reactive in a folded G4 because of the two K^+^ ions closely associated with the central tetrad that might disfavor formation of G^•+^ at this site. Importantly, one should note that any Gs that are extruded from the double helix or the quadruplex are particularly reactive toward oxidants because of solvent accessibility (38,45). Many promoter G4s have an excess number of Gs; that is, some but not all tracks have four or five Gs instead of three, such that they will reside in loops of the folded G4 (Table 1) (45). For example, observation of an oxidation pattern of 5′-GGGG-3′ would suggest that the first three Gs are part of a G4 and the 3′-most G is looped out and reactive. Other ROS, such as singlet oxygen and ozone, do not follow a sequence pattern but rely entirely on the accessibility of the π face of G toward reaction (46). Indeed, we used this feature with G4s of known structure to suggest the absolute configuration of chiral oxidation products, such as the hydantoins Sp and Gh (see Figure 3) (47). Sp and Gh are further oxidation products of OG (48,49), present in lower amounts than OG in cells undergoing oxidative stress, that arise because OG is thousands of times more reactive than G toward one-electron oxidation and many other ROS as well (50). Many other oxidation products exist besides OG, Sp and Gh, and these have been reviewed (39,51); however, we will focus on OG for the remainder of this discussion.

Oxidized base lesions such as OG are typically repaired by the base excision repair (BER) pathway (Figure 3B), and for OG this is a multilayer process (52). OG itself is not very mutagenic because it is well accommodated in the B-form helix and base pairs well with C (53). However, its potential mutagenicity lies with the ability of OG in single-stranded DNA (ssDNA) to rotate about the glycosidic bond from the *anti* to the *syn* conformation, alleviating some unfavorable interactions between the C8 carbonyl and oxygen atoms of the deoxyribose–phosphate backbone. OG_syn_ forms a good base pair with A during replication, which would lead to a G→T transversion mutation if not repaired (54). Thus, the BER glycosylases that break the glycosidic bond between sugar and base are highly specific in the case of OG for double-stranded (dsDNA) DNA substrates. OGG1 is a glycosylase that recognizes and removes OG when paired with C; MUTYH correspondingly recognizes OG paired with A and removes the unmodified, but incorrect, A (52,54). After repairing the OG:A mispair, the OG:C base pair can then undergo normal repair by OGG1 in a second round. Accordingly, cell lines or mice with Ogg1 knocked out are viable and do not have a prominent change in phenotype, but are somewhat more susceptible to inflammatory stress (55). In contrast, mutations in MUTYH can lead to colon cancer because the OG:A mispair is poised for mutagenesis (56).

Excision of incorrect nucleobases by BER glycosylases leads to the formation of an abasic site, or apurinic (AP) site in the cases of OGG1 and MUTYH (52,54,57). Some glycosylases can also remove the sugar fragment by β- and δ-elimination reactions, but for OGG1 this reaction is slow; instead, the very toxic abasic site is likely handed off *in vivo* to AP endonuclease 1 (APE1) for strand scission at the 5′ phosphodiester bond adjacent to the AP (58). APE1 is a much more abundant protein in cells than BER glycosylases, and it serves multiple functions, as discussed below. Repair is completed after removal of the sugar fragment by dNTP insertion into the gap of the duplex by polymerase β (POLB) and ligation of the nicked strand.

Other oxidized lesions such as the hydantoins Sp and Gh can follow a similar repair pathway, although the glycosylases involved are the NEIL1–3 family that can process a wide variety of oxidized base lesions other than OG (40). Hydantoins are the best substrates studied so far, but unlike OGG1, there is no strong preference for the base opposite, or even a requirement that the lesion be part of a duplex (59–62). Interestingly, Sp and Gh in the context of a G4 are particularly good substrates for NEIL3, a monofunctional glycosylase that also requires APE1 to complete strand scission (45,61,63). Unlike OG, the hydantoins are all duplex-destabilizing lesions, as is the AP site (53). This has broader implications when the lesion is part of a PQS, and in fact, the process of DNA damage and repair might explain an energy conundrum with promoter G4s. These GC-rich sequences should be very stable as Watson–Crick base-paired duplexes. What is it that triggers strand separation and refolding to a G4 and perhaps also an i-motif (1)? Typically, i-motif folding may require a drop in pH below 7 (64), but why would a G4 want to fold? In terms of the duplex–quadruplex equilibrium, the duplex should always be more stable because of a larger number of base pairs and strong π stacking, although supercoiling and protein binding could impact the equilibrium (20).

**Figure 4.**
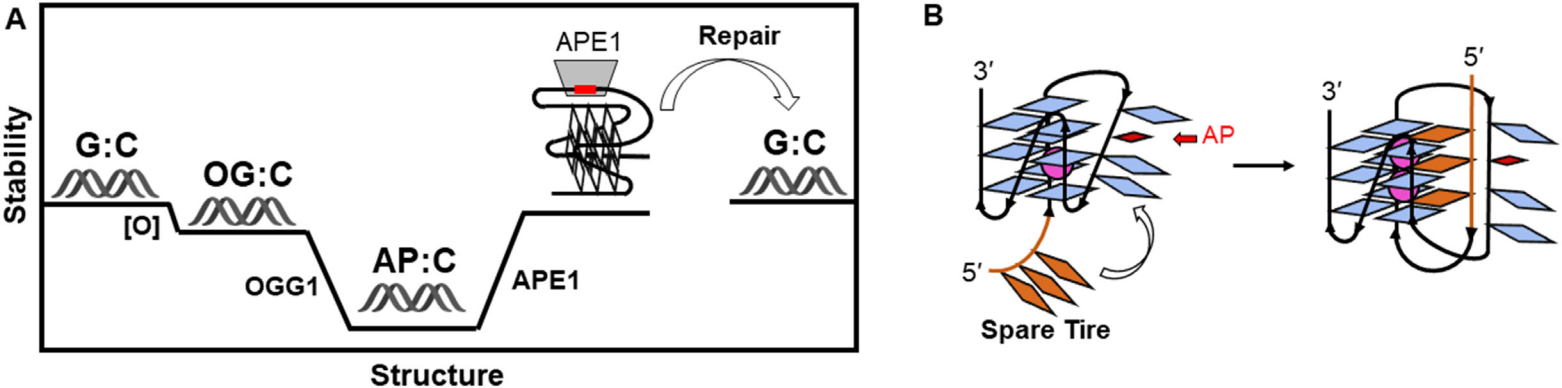
(**A**) Schematic drawing of stabilities, roughly equating to *T*_m_ measurements, for G:C base pairs versus lesions in various contexts. The AP site has low stability in a duplex context but can be refolded to a stable G4 if the AP site is looped out. (**B**) A fifth track, or ‘spare tire’, can be swapped for the damaged track to generate a stably folded G4.

Consider the energy diagram of Figure 4A. The G:C duplex represents a PQS present in a double-helical context that is presumably of very high stability because it is GC rich, and this is typically measured in oligonucleotides as a thermal melting temperature, *T*_m_ (45,53). Introduction of OG into the sequence will only destabilize the duplex slightly, by ∼2–3°C in *T*_m_. The DNA repair pathway initiated by OGG1 leads to an AP that is highly destabilizing to the duplex. In general, either OG or AP would also be destabilizing to a quadruplex structure (31,45). However, as noted earlier, essentially all promoter PQSs have excess Gs. Either there are four or more Gs in some of the tracks or, more commonly, there are more than four G tracks. In the case of a base lesion, the G4 can still assemble by either sliding a track to a GGG run or by looping out the damaged track and swapping in the fifth track, or ‘spare tire’ (Figure 4B) (45). Essentially every promoter G4 that has been characterized, as well as the hTelo (65), has the ability to accommodate a damaged base either by sliding or by swapping.

Evidence that the ‘spare tire’ mechanism works has been seen for several G4s (30,45,66). NMR characterization of these dynamic processes in *c-MYC* indicates that refolding of a stable G4 is quite slow, on the order of many minutes to hours (67). Thermal denaturation studies of AP-containing G4s show that wild-type stabilities are achieved when a fifth track is engaged and the AP is looped out (30,31). If the AP is forced to reside in a core position of the G4, *T*_m_ values are 15–20°C lower, which is about the same degree of destabilization as an AP in a duplex. When repair is complete, the stability of the duplex returns to its original high level.

This picture helps explain how DNA damage and repair could collaborate with the G-rich sequence to facilitate G4 folding. This would imply that G oxidation and repair by OGG1 plus APE1 provides a mechanism to reveal the folded G4, dissociating the template and non-template strands, and assisting with transcription initiation. As we detail below, both OGG1 and APE1 provide additional benefits to gene induction by serving as sites for binding of activating transcription factors, including NF-κB, HIF-1α, AP-1 and others.

### G4s, oxidative stress and gene expression

From the point of view of DNA damage and genome integrity, placement in key regulatory sites of poly-G tracks that have a high potential for oxidative damage and mutations is a poor evolutionary choice. However, if we assert that OG is not highly mutagenic because DNA repair is efficient, then use of OG as a transient epigenetic mark in a PQS makes sense (4). The refolding of the promoter PQS upon BER processing of OG to AP provides a switching mechanism to recruit new proteins to the folded G4. A recent analysis described G4s as ‘hubs’ for binding of transcription factors (68); they might also be thought of as bus stops where DNA processes pause to let different passenger proteins on and off the bus.

Evidence of a correlation between oxidation in promoters and increased gene expression was found more than a decade ago when Avvedimento and colleagues found increases in OG (via ChIP-PCR studies for OGG1) during estrogen- or retinoic acid-induced expression of BCL2 (69,70). Interestingly, their studies pointed to H_2_O_2_-induced oxidation of G during LSD1-mediated demethylation of histone lysines. Independently, Gillespie and coworkers showed that the G4-containing promoter of *VEGF* was oxidized during oxidative stress-induced upregulation of the gene (71,72). The proximity of the G4 in *VEGF* to the TSS suggested a role for OG in this sequence that was known to impact gene induction, although the sequencing studies for OG were performed at 0.5 kb resolution.

**Figure 5.**
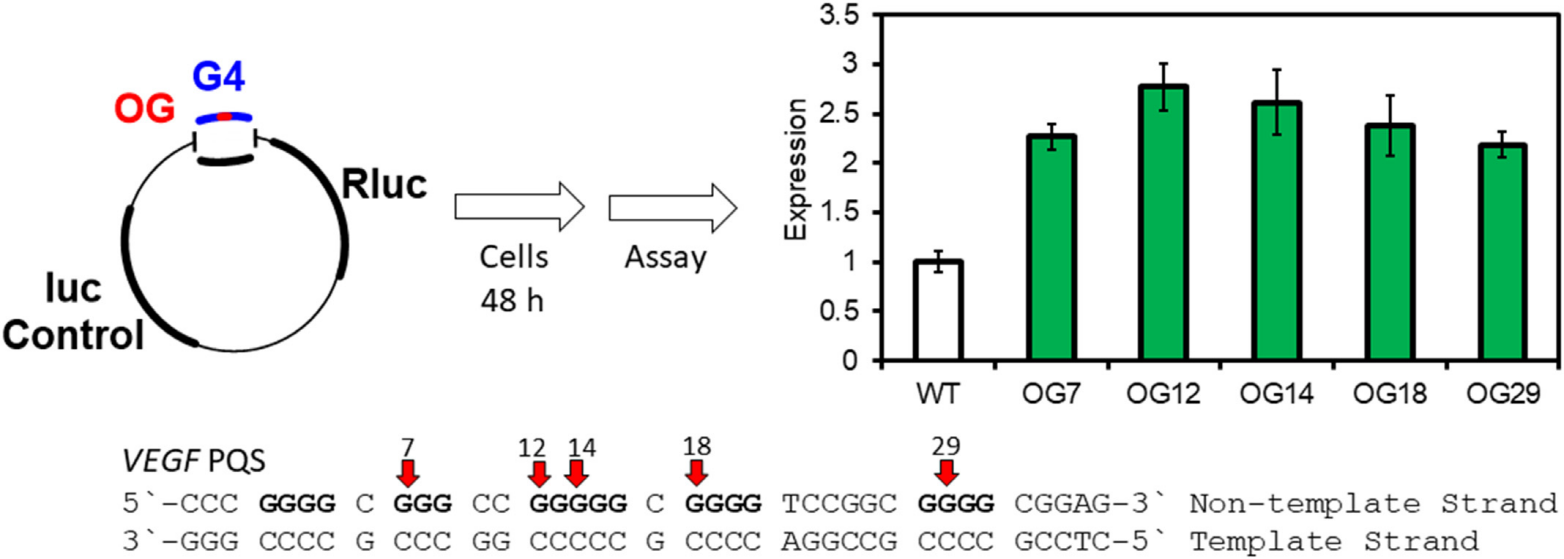
A dual-luciferase plasmid was manipulated to have an OG-containing PQS replacing the TATA box of the promoter. Transfection into cells and analysis after 48 h showed robust increases in gene expression (31).

We therefore asked whether we could recapitulate the increase in gene expression observed with G oxidation in a molecularly defined reporter system in cells (31). We chose to manipulate DNA plasmids synthetically so that we could control the precise location of DNA modifications and their sequence location at the same time as monitoring gene expression under different cellular conditions. The plasmid system we chose was a dual-luciferase plasmid with the SV40 promoter controlling the *Renilla* luciferase (*Rluc*) gene and the HSV-TK promoter ahead of the firefly luciferase gene (*luc*, Figure 5). Both promoters have TATA boxes at ∼25 nt upstream of the TSS in the non-template (or coding) strand. We replaced one of the TATA boxes with a PQS leaving the other promoter as ‘wild type’ to serve as an internal standard. Next, after growing sufficient quantities of plasmid, we used nicking endonucleases to remove a portion of the G-rich strand containing the PQS and to ligate in the same oligomer but with a single modification, for example either OG or F at a precise location. The latter, F, is an AP analog that is more chemically stable than AP but still a good substrate for APE1 during BER (73).

In the first study, we placed an OG or F modification at one of five different positions in the *VEGF* G4 sequence; some of these positions were in presumed loops of a folded G4 and others were in core positions that would require the engagement of the fifth track to form a stable quadruplex; thus, both four-track and five-track versions of the *VEGF* G4 sequence were studied (31). Transfection into mouse embryonic fibroblasts (MEFs) of the OG-modified plasmid compared to normal G-containing plasmids could be compared in WT MEFs, OGG1^–/–^ MEFs and WT MEFs with APE1 siRNA knockdown. Typical results are shown in Figure 5.

**Figure 6.**
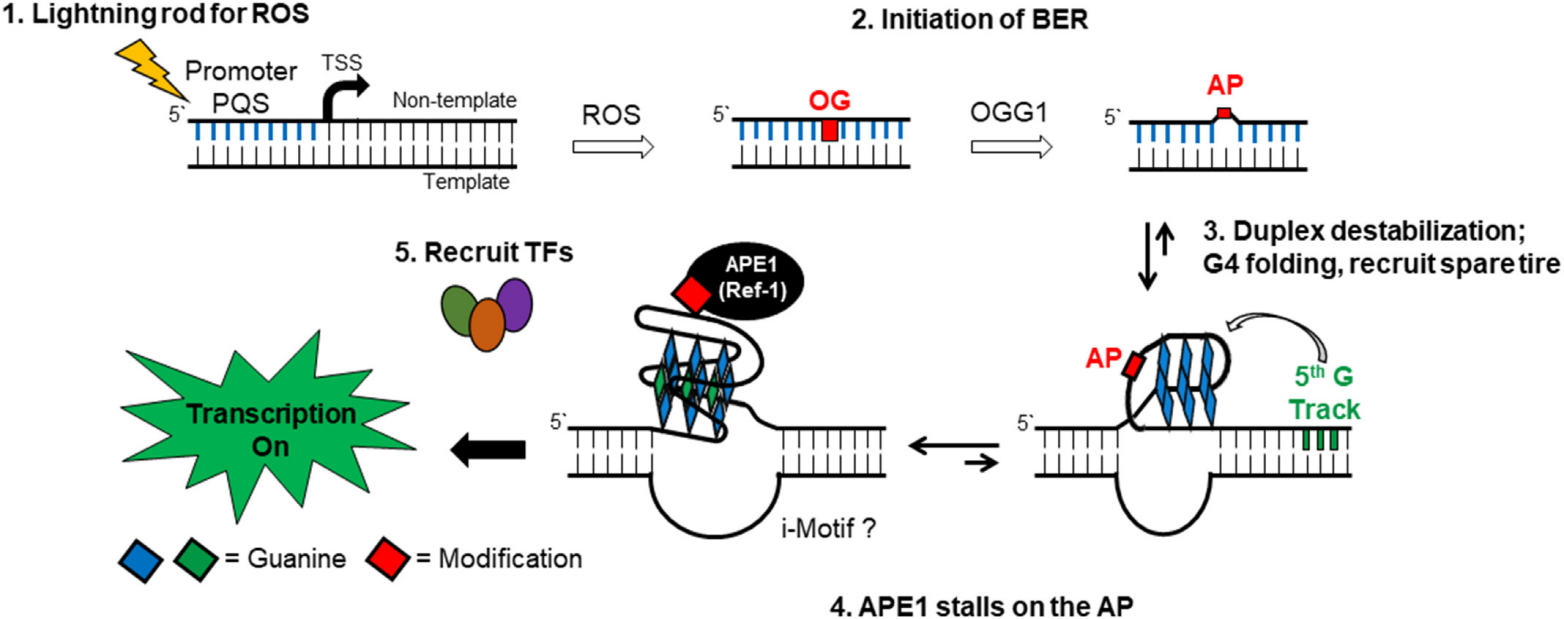
Proposed mechanism for upregulation of genes with oxidative damage in their promoter G4. The PQS acts as a sensor of oxidative stress; subsequent BER refolds the sequence to a G4 at which APE1 is bound and can recruit activating transcription factors.

Several conclusions can be drawn from these and subsequent studies performed with the G4 sequences from promoters in *NTHL1* and *NEIL3* (BER glycosylases) (30,31), *PCNA* (repair processing) (32) and *RAD17* (damage response) (33): (1) A G4 sequence containing DNA damage in the non-template strand of the promoter increases gene expression by ∼3-fold for OG or ∼5-fold for AP. (2) The exact position of the damage in the G4 is not very important. (3) The fifth track enhances the effect, either by ensuring G4 folding or by assisting dynamics to find the best fold. (4) The BER pathway is required—OGG1 is needed to convert OG to AP and APE1 is absolutely required to see the increase in gene induction. (5) The magnitude of the effect is somewhat cell line dependent; in addition to MEFs, several human cancer cell lines were studied (11). (6) Even without OG or AP, a PQS in the promoter could increase gene expression under conditions of oxidative stress induced by addition of tumor necrosis factor α (TNFα) to the cell medium (30). TNFα leads to higher expression of iNOS, which generates peroxynitrite and ultimately carbonate radical anion (74). The finding of increased gene expression is consistent with the conclusion that oxidized lesions generated during endogenous oxidative stress are giving the same response as those specifically inserted by oligonucleotide synthesis. An overall mechanism is proposed in Figure 6.

As discussed in the earlier sections, endogenous oxidative stress generates CO_3_^•−^ that leads to selective reaction at poly-G sequences such as a PQS. These sites become a focal point for DNA damage in which long-range charge transport will relocate an electron hole to these sites (4,41). If a promoter PQS is oxidized at any one of multiple sites in the sequence, the DNA duplex is still stable (53). However, initiation of BER by the action of OGG1 leads to the destabilizing lesion AP (52,53). Like many proteins in the repair pathway, OGG1 binds tightly to the AP product, but then hands off to APE1 for the next step, strand cleavage (58). APE1 is the centerpiece of many studies on the impact of DNA repair on gene expression because it serves multiple functions: BER nuclease, transcription factor recruiter and G4 binder (75–77). When APE1 binds to AP in a G4 context, the nuclease function is paused, and this pausing allows the redox effector factor function of APE1 to recruit activating transcription factors, including HIF-1α, AP-1 and others (30,72,75).

Overall, the mechanism in Figure 6 proposes that G4 sequences in promoters are sensors of oxidative stress that act as an antenna for DNA oxidation. When OG is formed, the BER pathway begins with OGG1 creating the duplex-destabilizing abasic site AP. The AP site may be better accommodated in a folded G4 in which the AP is extruded into a loop; this is the switching point from repair to gene induction, because APE1 binds to G4s but its nuclease activity pauses. Once the repair bus has stopped at the G4 bus stop, APE1 facilitates gene expression through transcription factor binding (4).

This proposed mechanism is not the only one involving G oxidation and gene expression. In related work, Boldogh and coworkers showed that the transcription factor NF-κB was recruited to OGG1 sites during oxidative stress (78,79). Xodo and coworkers found *KRAS* upregulation after oxidation of G to OG in a promoter PQS was acted upon by the BER machinery to recruit activating protein factors (66). Tell and coworkers found that transcription was initiated when OGG1 sites were processed in the SIRT1 promoter to allow buildup on APE1, Ku70/80 and RNA pol II for gene induction (80). Hanawalt (22), Myong (23) and others point to modulation of gene expression by R loops, and the ability to form G4s impacts the stability of the R loop. Furthermore, it is well known that DNA damage on the template strand can impact gene expression because the DNA repair machinery should be recruited to TSSs in advance of RNA synthesis, i.e. transcription-coupled repair (81). However, it is the gene activation mechanism involving DNA damage on the non-template strand that has intrigued many laboratories in the past few years. Central to this appears to be the ability of BER proteins to recruit transcription factors as passengers on the bus to gene expression (4,79,82).

### APE1 drives the bus

**Figure 7.**
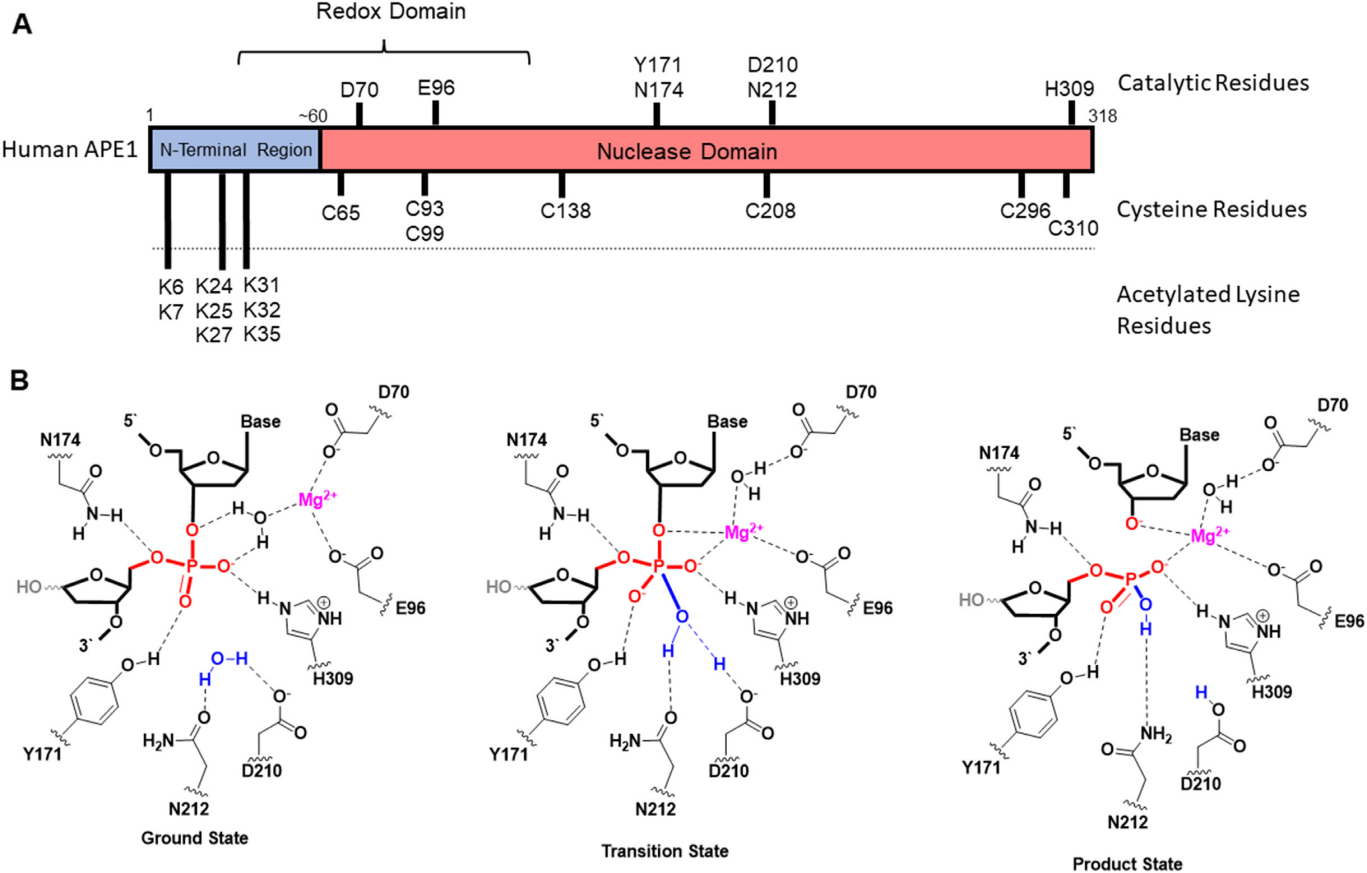
Key amino acids in the APE1 protein essential for its endonuclease activity, REF abilities and post-translational regulation. (**A**) Schematic of human APE1 to draw attention to key catalytic amino acids based on a solved structure (96), as well as cysteine and acetylatable lysine residues. (**B**) Proposed mechanism for APE1-catalyzed hydrolysis of a phosphodiester bond 5′ to an AP site as previously reported (96). The structure was studied with the tetrahydrofuran (F) analog of the AP (96). For clarity, the 1′-OH on the baseless sugar was added to the mechanism in light gray to display the *in vivo* substrate.

What chemical and biological features of APE1 enable this protein to drive the bus that coordinates gene regulation under oxidative stress conditions? Mammalian cell studies identified APE1 as a high copy number protein (∼10^4^–10^5^ copies/cell) with a long half-life (∼9 h) found in nearly all cell types that is essential for cellular survival (77,83,84). The biological importance of APE1 was established when embryonic lethality was observed in Ape1^–/–^ knocked out mice, while heterozygous mice (Ape1^+/–^) were found to thrive early on but displayed an increased apoptotic response to oxidative stress, and a high incidence of cancers later in life (85–87). The protein is found in the nucleus, cytosol and mitochondria, for which the distribution can change in some cancers (88–90). The human APE1 protein is 318 amino acids long, has a nuclease domain from positions 60 to 318 homologous to *Escherichia coli* exonuclease III and has an N-terminal region of ∼60 amino acids (Figure 7A) (77,91). The first ∼40 amino acids are unstructured, and the amino acids between ∼40 and 60 are structured in solution (92). In humans, APE1 possesses a variety of functions on DNA that include an AP endonuclease, 3′,5′ exonuclease, 3′ phosphatase and a nucleotide incision repair catalyst; in RNA, APE1 has RNase H activity and functions in RNA metabolism; and lastly, the protein is a redox effector factor (REF1; positions 30–127, Figure 7A) for regulation of gene expression (76,93–95). The latter property of APE1 is so vital to cellular processes that the complete descriptive name for the protein is APE1/REF1 (95). Thus, APE1 is critical to cellular survival, and how this protein achieves this wide variety of functions has been a long-standing research question that remains an active area of study (76).

How APE1 can guide protein binding on DNA during oxidative stress in which both the BER and gene regulatory features of the protein are needed requires closer inspection of the chemistry, which will be the focus of this section. For readers interested in the other activities of APE1 that include its exonuclease and RNA processing properties, there exist excellent reports on these topics (94,97,98). Abasic site substrates for APE1 are provided by monofunctional glycosylases that hydrolyze the glycosidic bond of chemically modified nucleotides to yield the AP (Figure 3B) (52). As an endonuclease, APE1 is proposed to scan the DNA by diffusion along the strand with a residence time of 2–3 μs per base pair until it binds the AP in DNA (99); then, APE1 hydrolyzes the 5′ phosphodiester of the AP site yielding a 5′ fragment with a 3′ hydroxyl group, and a 3′ fragment with a 5′-deoxyribose phosphate (5dP; Figure 3B) (96). Structurally, APE1 engages an AP by flipping it into an active site binding pocket that kinks the DNA by ∼35°, leaving an orphan base in the site opposite the AP in the complementary strand that is stabilized by protein interactions. There are many important active site residues identified by biochemical, structural and computational analyses (86,100–102). Noteworthy residues are Asp210 that is essential for activation of the H_2_O that attacks the phosphodiester to yield the pentavalent intermediate, as well as Asp70 and Glu96 involved in the coordination of the Mg^2+^ cofactor needed for substrate binding, transition state stabilization and product binding (Figure 7B). Arginine 177 stabilizes the APE1–AP DNA-bound structure via intercalation to slow product release and allow the next enzyme to complete the repair process (83). In duplex DNA, APE1 binds the product to allow a handoff to POLB as the next member in the BER process that prevents the release of the more genotoxic lesion, a single-strand break (73,83,103). POLB has both lyase activity to remove the 5dP and polymerase activity to select the correct dNTP directed by the nucleotide opposite the AP site (104). Finally, ligase seals the nick to yield the repaired product (52). This provides a clear picture of how the enzyme is proposed to function as an endonuclease on a canonical substrate in short-patch BER.

Enzyme activity assays for APE1 have found that the cleavage is most robust for AP-containing dsDNA and shows product inhibition as discussed earlier. In solution, APE1 favors endonuclease activity at slightly alkaline pH (7.4–7.8) and near-physiological concentrations of K^+^ (50–140 mM) and Mg^2+^ ions (>2 mM) (105,106). Initial studies found APE1 can cleave an AP in ssDNA, small dsDNA bulges, model replication forks, transcription bubbles, R loops and DNA/RNA hybrid duplexes (106,107). The activity of APE1 toward AP-containing G4s found in the *c-MYC* (108), hTelo (61,75,109), *VEGF* (110) and *NEIL3* (30) sequence contexts has been analyzed to find reduced activity compared to dsDNA substrates. In the hTelo, *VEGF* and *NEIL3* G4 studies, the position of the lesion impacted the endonuclease cleavage yield; additionally, in the *VEGF* and *NEIL3* G4 studies whether the fifth G run was present or not impacted the APE1 cleavage yields. The efficiency and rate of APE1 cleavage of an AP in G4 contexts display differences in the dependence on the concentration of K^+^ ions compared to dsDNA (61,75). In general, the enzymatic activity was nearly abolished as the concentrations of K^+^ approach physiologically concentrations. This may have an important role in preventing APE1 from cleaving an AP when a complementary strand is not present that would be necessary to permit the handoff to POLB. In the proposal that G4 folds serve as genomic stops for APE1 to coordinate movement of protein transcription factors, the reduced activity supports this claim, but binding must be retained at the higher ionic strength.

The binding of APE1 to AP or an F analog in dsDNA has been analyzed by a variety of methods, under similar but not identical conditions, and on different sequence contexts and nucleotides opposite the F site (111–114). The key observation is that APE1 binds an AP (F)-containing dsDNA with a low nanomolar dissociation constant (*K*_D_). The N-terminal region of APE1 when removed has a minor impact on the binding interaction with dsDNA; however, the activity of APE1 increases when the N-terminal region is removed as a result of decreased product inhibition suggesting the region is involved in the transfer of the product to the next BER member POLB (115–117). In G4 contexts, the catalytically inactive D210A APE1 mutant was first demonstrated to bind the *c-MYC* parallel-stranded G4 in 50 mM K^+^ based on a supershift on a native gel; a binding constant was not determined in this study (108). The binding of wild-type APE1 to the wild-type hTelo sequence in the presence of 150 mM Na^+^ ion studied by SPR (surface plasmon resonance) provided a 30 nM *K*_D_ value (75). It is worth noting that Na^+^-bound hTelo G4s adopt basket (antiparallel) topologies (118). Further, the observation of APE1 binding to the Na^+^-bound hTelo G4 was the first to identify APE1 binds non-lesion-containing G4 folds (75). A recent study confirmed wild-type APE1 binds native and F-containing hTelo G4s in 140 mM K^+^ ion-containing solutions (109), where the native G4 adopts a hybrid fold and the lesion-bearing G4s display folds not fully characterized (61,109); furthermore, the binding was location dependent (109). In our studies, wild-type APE1 bound the four- and five-track *VEGF* G4s that favorably adopt parallel folds in K^+^ solution, and there was dependence in the *K*_D_ value on the location of F and whether the fifth G track was present or not (119). Lastly, the binding of APE1 to hTelo and *VEGF* G4s was found to be highly dependent on the presence of the N-terminal region (75,119), a sharp deviation from dsDNA binding by this protein. This final observation suggests that G4 binding through the disordered N-terminal region may be strong. Analysis of tryptophan fluorescence changes when APE1 engages its substrate found large structural deviations for binding to F-containing dsDNA and small deviations for F-containing G4s (109), which might be explained by binding to the non-canonical folds via the disordered N-terminal region where tryptophan is not found in the sequence.

A few critical conclusions can be drawn from the binding studies: (1) Wild-type APE1 binds lesion-containing G4s coordinated to K^+^ ions. (2) Binding of G4s by the N-terminal region of APE1 helps address the poor enzyme activity observed for AP-containing folds. (3) Wild-type APE1 binds native G4 folds adopting basket, hybrid or parallel topologies. This final point is interesting because folded genomic G4s were recently found to be hubs for transcription factor binding essential for gene regulation (68). Thus, these studies demonstrate that APE1 can stop the bus (i.e. bind) at G4 hubs to allow the exchange of proteins to coordinate gene expression. Whether the AP lesion is needed for APE1 binding *in cellulo* is not currently known.

One more unique property of APE1 is its REF activity to regulate downstream transcriptional activity by controlling the binding of critical transcription factors (120–122). This feature of APE1 is essential to cell growth (76) and is a promising drug target for the treatment of many different types of cancer (122). Transcription factors controlled by APE1 required for the response to oxidative stress include AP-1 (123), HIF-1α (72), NF-κB (78), p53 (124) and STAT3 (125). The N-terminal region of APE1 is critical for redox regulation, wherein C65 is the most important residue for the regulation (Figure 7A) (126). One proposal is that C65 and C93/99 function in the thiol–disulfide exchange reaction to reduce target disulfide-containing dormant transcription factors to sulfhydryls to activate them for gene regulation (126,127). Two challenges to this proposal exist. First, the cysteine residues (C65 and C93 or C99) are too far apart in the crystal structure to rationalize chemically a disulfide bond in APE1 (83,96); second, only C99 and C138 are solvent exposed, while the critical C65 is buried within the protein (96). A solution to this chemical challenge is the observation that APE1 is highly dynamic in solution allowing disulfide bond formation to occur (128,129). Similarly, APE1 could form an intermolecular disulfide bond with the transcription factor to aid in DNA binding and gene regulation, thus avoiding the complication of disulfide bond formation within APE1. Alternatively, a recent report proposed that APE1 induces a change in the target DNA conformation to enable transcription factor loading, and therefore does not invoke the REF1 domain in APE1 for disulfide-exchange chemistry (130). More studies are needed to better address whether these represent similar or distinct phenomena for gene activation.

Gene regulation during oxidative stress via G4 hubs and APE1 binding as a means to select transcription factor partners to induce mRNA synthesis are supported by prior work. This occurs as a result of preferential G oxidation in G-rich human promoters to localize the BER proteins, in which APE1 can bind the lesion-containing G4 folds using the N-terminal region of the protein. Transcription factors will then be recruited to turn transcription on, and then eventually the complex will dissociate to allow completion of the DNA repair process, returning the promoter to the native dsDNA state. The finding that APE1 can bind G4s without a lesion may provide an additional mechanism for gene induction facilitated by APE1. Future work is needed to address this point. The N-terminal region of APE1 possesses critical lysine residues that are post-translationally modified with acetyl groups (Figure 7A). The acetylation status of the protein is another layer of modifications used to fine-tune the protein–protein interactions of APE1 (116,131), and this has been proposed as a component of the APE1–G4 interaction responsible for gene regulation (132). Studies with other DNA repair proteins such as OGG1 have identified cysteine oxidation as an additional post-translational modification used by cells for regulation when the protein switches between DNA repair and gene regulation (79,133). Interestingly, APE1 has seven cysteine residues that can be oxidized (Figure 7A), a few of which were found to be oxidized in cells (134), and how this alters the APE1–G4 interactions has yet to be addressed (Figure 7A). The knowledge that APE1 binds G4s requiring the N-terminal region, but the catalytic activity is attenuated, suggests a possibility that the protein can bind the G4 through an alternative conformation. Future experiments to understand the full scope of post-translational modifications to APE1 responsible for switching the protein from repair to regulation and structural analysis of APE1 binding G4s are needed.

### Location, location, location

**Figure 8.**
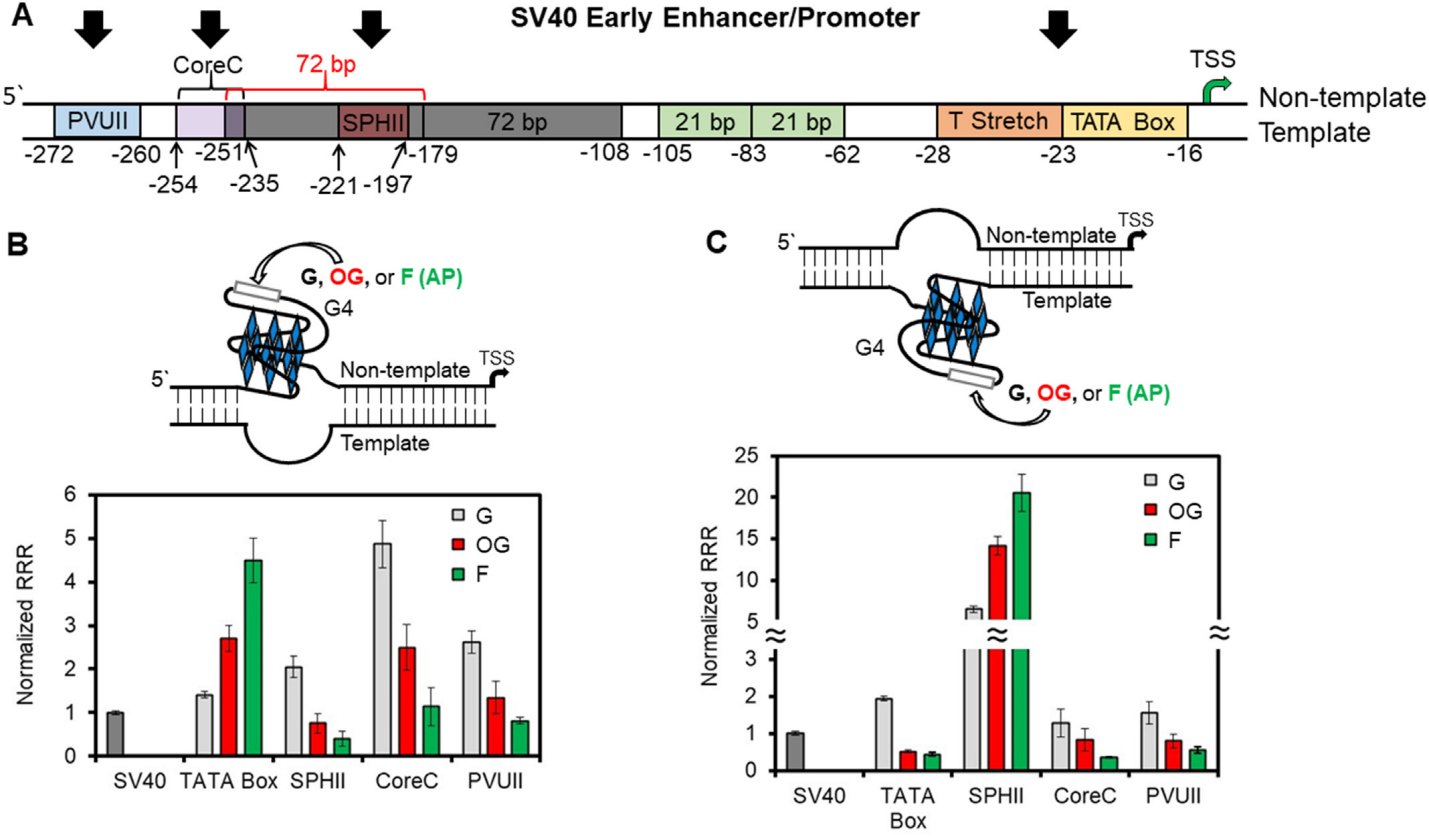
The *VEGF* PQS was moved through the SV40 promoter on both strands with the native G or synthetically installed OG or F to analyze the impact on gene expression. (**A**) Schematic of the SV40 promoter and key elements. Gene expression changes were observed when the G-rich strands with and without OG or F were synthetically installed on the (**B**) non-template or (**C**) template strand at locations indicated by arrows in panel (A) (11). In panels (B) and (C), the *x*-axis refers to the promoter element replaced with the PQS and the *y*-axis is the relative response ratio (RRR = Rluc/luc) normalized to the SV40 promoter expression. The Rluc promoter was the one replaced with the PQS.

The discovery of gene regulation guided by an APE1–promoter G4 interaction was made on a sequence found close to the TSS on the non-template strand (Figure 8A) (31). Human promoters are known to have significant GC skew correlated with elevated gene expression (135). As the GC skew increases, the probability of non-canonical structures such as G4s and the propensity for R-loop formation increase (4,21). The non-canonical structures such as R loops and possibly G4-forming sequences have been proposed to play a key role in gene regulation (4,21,135). Research in our laboratory in the model SV40 promoter with an embedded *VEGF* PQS without a lesion studied in mammalian cells found that the presence of the G-rich sequence increased the promoter strength (Figure 8B and C, bars labeled G) (11). The magnitude of enhancement was dependent on the location or distance from the TSS and the non-template versus template strand of occupancy for the G-rich sequence. Generalizations to human promoters are not direct because other factors in the genome are at play; nonetheless, this observation demonstrates that a PQS can influence gene expression likely by an increase in GC skew for recruitment of activating transcription factors.

As described earlier, amplification of this activation effect by oxidative modification of a G nucleotide to OG or its repair intermediate AP (F) was demonstrated for the *VEGF*, *NEIL3*, *NTHL1* and *PCNA* PQSs near the TSS on the non-template strand with dependence on APE1 (Figure 8B) (30–32). Does the same impact on gene expression occur when the construct is flipped over to the template strand of the promoter at the same site? Studies with the *VEGF* PQS containing OG or F at this site revealed that transcription was turned down 3-fold (Figure 8C) (136). A similar observation was found for the *RAD17* PQS containing OG near the TSS on the template strand (33). Through the utility of siRNA knockdown experiments, the gene deactivation was found to be dependent on the Cockayne syndrome B protein (CSB, aka ERCC6) (136); this is consistent with a prior study that found CSB is needed for resolving folded G4s to allow advancement of the transcriptional machinery (137). The CSB protein interacts with the RNA pol II complex to aid in sensing and resolving DNA damage via the transcription-coupled repair pathway (138), which suggests the presence of a damaged PQS could fold to a G4 to engage CSB and stall transcription. In contrast, when a non-modified PQS was in the same location gene expression was not CSB dependent, and therefore likely the sequence failed to adopt a G4 fold, and transcription continued unabated (136). Many mysteries still exist regarding the role of CSB in gene deactivation. (1) The CSB strongly binds intermolecular G4s but has a low affinity for intramolecular G4 folds (139), in which the latter is the likely fold in the studies described. (2) Is the function of CSB to resolve the lesion-containing G4 fold, recruit the transcription-coupled repair machinery or is CSB needed to achieve both functions?

Computational inspection of the human genome for PQSs found that they are enriched in promoters with the distribution centered at the TSS and expanding outward in both directions by ∼1000 nt (17,18). With this knowledge, our laboratory moved the OG-containing *VEGF* PQS to other promoter positions in either strand to observe the impact on gene expression (11). In the positions studied, gene activation was observed for OG in the PQS context at two locations: the first near the TSS on the non-template strand as previously described, and the second on the template strand near position −200 (Figure 8B and C). All other positions found OG in the PQS context turned transcription off. The most important takeaway from these studies is that promoters are like real estate, and it is all about location, location, location! Experimentally, this means that studies to understand which oxidation events in a promoter PQS result in activation versus those that lead to deactivation will need to be on a case-by-case basis.

The other genomic element around TSSs where PQSs are enriched is in 5′-UTRs (17,18). Examination of how oxidative modification of G in the PQS in 5′-UTRs impacts gene expression has yet to be examined. The 5′-UTR differs from the promoter in that the non-template strand PQS in the genome is transcribed into the mRNA transcript. For non-oxidized PQSs, studies have found that when these G-rich sequences reside in the non-template strand of the 5′-UTR, the transcriptional output of the gene is enhanced (23). The proposal for the increased expression is the formation of a transcription-induced R loop stabilized by a G4 fold that results in successive rounds of efficient transcription. The regulation of transcription by R loops has been reviewed (21,22). Whether oxidation of G in the non-template strand PQS when located in the 5′-UTR will have an impact on gene expression is not known, although binding of the AP-containing G4 by APE1 could significantly stabilize the G4 fold and impact expression. Oxidation of G to OG in the non-template strand (i.e. coding strand) of a 5′-UTR outside of a PQS context drove downregulated transcription (57). As for the template strand, G oxidation to OG in a non-PQS context stalls transcription because of initiation of transcription-coupled repair (140,141), and therefore, it is anticipated that G oxidation in a PQS context in this strand will also downregulate gene expression. In situations where RNA pol II can read through the template OG-containing PQS, the OG heterocycle can be read as a T nucleotide via the Hoogsteen face to direct misincorporation of A in the transcript (142).

### Critical ties to cancer

Critical components of cancer are DNA damage, DNA repair and aberrant gene expression. A common example of DNA damage and its ties to cancer is G oxidation to OG. The OG base when located in a DNA template strand can direct insertion of dCTP opposite via pairing with the Watson–Crick face or dATP by pairing on the Hoogsteen face during replication (143). In the event of dATP insertion and no repair, a second replication event would result in a T:A base pair in one daughter strand where there was originally a G:C base pair (i.e. G→T transversion). The frequency of OG causing G→T transversions is low in DNA repair-competent cells (∼0.1%) (144). Hyperoxidation of G to Sp or Gh in template strands yields a mixture of G→T and G→C transversion signatures as a consequence of the hydantoins pairing with either purine 2′-deoxynucleotide triphosphate leading to nearly 100% mutation (145,146). Long-term exposure to oxidative and inflammatory stress has shown significant levels of G→T and G→C transversions in the genome in support of DNA damage as a mechanism for cancer initiation and progression (3). Definitive demonstration that OG, Sp/Gh or any other damaged G nucleotide is the origin of these mutations has yet to be reported.

BER pathways for DNA damage associated with cancer are dependent on the protein. The glycosylase MUYTH is responsible for initiating repair of an OG:A base pair, and when the enzyme is mutated to an inactive state, a high incidence of colon cancer is observed (54,56). Additionally, loss of APE1 is embryonic lethal; however, misregulation of APE1 and altered cell localization impact its repair and redox signaling capabilities in many cancers (86,87,122). The APEX1 gene is relatively small and is not highly mutation prone like some genes, but a few mutations have been noted in cancers that result in persistent genomic stress (147). One of many cellular pathways impacted by aberrant APE1 in cancer is the ability to respond to stress via binding to regulatory G4s as described earlier. An active area of cancer research is to drug APE1 via its nuclease abilities to increase the success of DNA-damaging chemotherapeutics, or alternatively to target the redox signaling of APE1 to interfere with gene expression in diseased cells (122). The situation is different for OGG1 and the NEILs. Mice that are Ogg1^–/–^ are overall normal, except they have 2-fold higher levels of genomic OG and are more sensitive to oxidative and inflammatory stress compared to wild-type mice (55,148). The stress sensitivity observed likely results from an inadequate stress response by the mechanisms outlined herein. Lastly, the Neil1/2/3 glycosylases have been knocked out of mice to find no predisposition for cancer and no increased frequency of mutations, but they may have some metabolic disorders (149,150). This suggests the NEILs are not backup enzymes for one another and have distinct cellular functions beyond canonical DNA repair. These observations highlight that BER proteins differ widely as to their impact on cancer predisposition.

Genome stability can be negatively impacted by folded G4s. On template strands, G4 folds can cause stalled replication progress causing replication-fork collapse resulting in double-strand breaks that are highly mutagenic and specifically repaired by non-homologous DNA end joining (151). Sequences in the human genome that can adopt highly thermodynamically stable G4s are sites of known somatic mutations and recurrent mutations in cancer genomes (152). Sequencing for G4s has exposed these sites to be associated with gene amplification events found in cancers (12,19). Targeting cellular G4s with small molecules is an approach proposed for the treatment of cancer by changing the expressional output of oncogenes (9,10). This approach to downregulate an oncogene has been best studied for the *c-MYC* promoter G4, in which the c-MYC protein is overexpressed in a majority of human cancers (20). However, small molecules targeting G4s usually bind indiscriminately in cells and could target the ∼10 000 G4s that are known to fold, as well as induce folding of some of the other 700 000 PQSs that reside in the human genome (12,19). Ligand-bound G4s represent roadblocks to polymerases, they disrupt protein binding to G4s and they can alter chromatin structure resulting in many off-target impacts to the cells treated with these compounds. A solution is to target G4s with sequence specificity to avoid, or at least minimize, off-target impacts when treating cancer via regulatory G4s (153). The function of oxidized G4s for stress response regulation in cancers is not known, and whether targeting this pathway will be successful awaits more research discoveries.

## CONCLUSIONS

At first glance, the collaboration of G4 folding, which inhibits the progression of polymerases, with DNA damage and repair pathways that may be mutagenic, to upregulate gene expression, appears to be an unlikely one—a sort of double negative. Yet, multiple lines of evidence suggest that Nature is using these two key properties of G-rich sequences, namely their sensitivity to oxidation and their propensity to fold to stable G4s, in a productive way (4). Formation of OG in a promoter PQS is likely if long-range charge transport can conduct the electron hole to these sensitive sites. Refolding of the sequence to a G4 is then facilitated by the repair process in which APE1 plays the critical role—pausing at the G4 to switch to a gene regulatory pathway versus completion of repair. In this sense, OG serves a temporary epigenetic function as a DNA base modification capable of up- or downregulation of gene expression via its protein readers APE1 and CSB (136). OG as an epigenetic mark is not passed on to future generations of cells, but the potential quadruplex sequence in a regulatory site is (154). From viruses to humans, there are many examples of G-rich sequences being highly conserved, despite susceptibility to G→T mutations if not repaired, underscoring the important roles played by G-rich sequences (154). The participation of APE1/REF1 in both avoidance of mutagenesis and upregulation of oxidative stress-related genes mirrors the importance of both pathways, mutagenesis and aberrant gene expression, in cancer (4,77,121,122). Genomic hubs comprised of G4s could be more versatile than interactions with BER proteins, and crosstalk with other DNA repair pathways for gene regulation (155).

The study of G4s and DNA oxidation and repair in gene expression is being carried out from many different perspectives, to the ultimate benefit of science. On the one hand, biophysical chemists and structural biologists are characterizing how G4s fold, how specific proteins interact with DNA and the enzymology of DNA repair (17,27,59,63,75,83,96,109). All of these are *in vitro* studies, and while they provide detailed pictures of a given biomolecule, said molecule is not in the cellular setting. For whole-genome, potentially live-cell approaches, scientists use both low-resolution methods such as fluorescence microscopy or ChIP-seq and related methods to image and sequence sites in chromatin and targeted methods that deliver designed small molecules to folded G4s (10,19,69,71). This latter approach provides information about biomolecules in their native setting, but can alter the natural status of the cell by shifting the duplex–quadruplex equilibrium or by displacing G4-binding proteins with tight-binding small molecules; thus, there are uncertainties in these approaches also. We and others have found that some of the middle ground between *in vitro* and *in cellulo* DNA biochemistry can be discovered by the use of plasmids from the chemical biology toolbox (20,31,156). These are typically ∼7000 base-pair circular DNA duplexes that can be site-specifically altered to contain essentially any promoter sequence and any chemically stable base modification at a defined site. Plasmids containing G4 sequences transfect well into many cell lines, and upon entry, they are packaged with histones and transported to the nucleus where they experience the same suite of DNA-interacting proteins as chromatin (157). A further advantage of plasmid studies is that hundreds of copies are introduced to the cell, providing a robust readout for gene expression studies. There are disadvantages, however; these include (1) the fact that the histone modifications in plasmid nucleosomes may not be native, (2) supercoiling could impact the formation of G4s differently in plasmids and (3) long-range interactions, such as those mediated by large loop formation with CTCF, would likely be missing. In sum, all of the different approaches help us paint the complex picture of gene expression and the role of DNA repair and G4s in this important process.
